# Supplementary material for: The Systems Biology Research Tool: evolvable open-source software
Source: BMC Syst Biol. 2008 Jun 29;2:55. doi: 10.1186/1752-0509-2-55 (PMC2446383; doi:10.1186/1752-0509-2-55)
Supplement: Additional file 1 — SBRT Archive. An archive of the current version of the Systems Biology Research Tool. [file 1752-0509-2-55-S1.zip › sbrt-1.4.0/doc/developers_guide/api/sbrt/kernel/math/package-summary.html]

sbrt.kernel.math


|  |  |  |
| --- | --- | --- |
| **Overview** \| **Package** \| Class \| **Use** \| **Tree** \| **Deprecated** \| **Index** \| **Help** | |  |
| **PREV PACKAGE**   **NEXT PACKAGE** | **FRAMES**    **NO FRAMES**     **All Classes** |  |


---

## Package sbrt.kernel.math

Provides classes and interfaces for creating objects relevant to mathematics.

**See:**
  
          **Description**

| **Interface Summary** | |
| --- | --- |
| **EquivTester<T>** | This interface is used to represent *equivalence testers*. |
| **LinearComb<V>** | This interface is used to represent linear combinations. |
| **LinearComb.Term<V>** | This interface is used to represent the terms of linear combinations. |
| **MathExpr<V>** | This interface is used to represent mathematical expressions. |
| **Polynomial<V>** | This interface is used to represent polynomials. |
| **Polynomial.Term<V>** | This interface is used to represent the terms of a polynomial. |
| **QuadraticExpr<V>** | This interface is used to represent quadratic expressions. |
| **QuadraticExpr.Term<V>** | This interface is used to represent the terms of quadratic expressions. |

| **Class Summary** | |
| --- | --- |
| **AbstractLinearComb<V>** | This abstract class is a skeleton implementation of `LinearComb`. |
| **AbstractLinearComb.Term<V>** | This class is a skeleton implementation of `LinearComb.Term`. |
| **AbstractPolynomial<V>** | This abstract class is a skeleton implementation of `Polynomial`. |
| **DoubleEquiv** | This class is used to compare double precision numbers for equivalence. |
| **HashLinearComb<V>** | This class is a concrete implemenation of `LinearComb`. |
| **HashLinearComb.Term<V>** | This class is a concrete implementation of `LinearComb.Term`. |
| **Interval** | This class is used to represent closed intervals of the form [α, β], where α ≤ β. |
| **IntervalComparer** | This class is used to compare vectors of intervals. |
| **IntervalComparer.Diff<V>** | Instances of this class are used to hold a variable and a pair of corresponding intervals. |
| **IntervalEquiv** | This class is used to compare intervals for equivalence. |
| **VectorComparer** | This class is used to compare vectors of double precision numbers. |
| **VectorComparer.Diff<V>** | Instances of this class are used to hold a variable and a pair of corresponding intervals. |

| **Exception Summary** | |
| --- | --- |
| **ConstraintViolationException** | This class is used to indicate a constraint violation. |
| **ExceededLimitException** | This class is used to indicate that a limit has been exceeded. |
| **IllegalBoundsException** | This class is used to indicate the existence of illegal bounds. |
| **InfeasibleSolutionException** | This class is used to indicate that no feasible solution exists. |
| **InvalidPointException** | This class is used to indicate that a point is invalid. |
| **MissingVariableException** | This class is used to indicate that a varible is missing. |
| **UnboundedVariableException** | This class is used to indicate that a variable is unbounded. |

## Package sbrt.kernel.math Description

Provides classes and interfaces for creating objects relevant to mathematics.

---


|  |  |  |
| --- | --- | --- |
| **Overview** \| **Package** \| Class \| **Use** \| **Tree** \| **Deprecated** \| **Index** \| **Help** | |  |
| **PREV PACKAGE**   **NEXT PACKAGE** | **FRAMES**    **NO FRAMES**     **All Classes** |  |


---
